# Functions of Learning Rate in Adaptive Reward Learning

**DOI:** 10.3389/fnhum.2017.00592

**Published:** 2017-12-06

**Authors:** Xi Wu, Ting Wang, Chang Liu, Tao Wu, Jiefeng Jiang, Dong Zhou, Jiliu Zhou

**Affiliations:** ^1^Department of Computer Science, Chengdu University of Information Technology, Chengdu, China; ^2^College of Information Science and Engineering, Chengdu University, Chengdu, China; ^3^Department of Psychology, Stanford University, Stanford, CA, United States; ^4^Department of Neurology, West China Hospital, Sichuan University, Chengdu, China

**Keywords:** adaptive learning, Bayesian modeling, fMRI, learning rate, reward

## Abstract

As a crucial cognitive function, learning applies prediction error (the discrepancy between the prediction from learning and the world state) to adjust predictions of the future. How much prediction error affects this adjustment also depends on the learning rate. Our understanding to the learning rate is still limited, in terms of (1) how it is modulated by other factors, and (2) the specific mechanisms of how learning rate interacts with prediction error to update learning. We applied computational modeling and functional magnetic resonance imaging to investigate these issues. We found that, when human participants performed a reward learning task, reward magnitude modulated learning rate. Modulation strength further predicted the difference in behavior following high vs. low reward across subjects. Imaging results further showed that this modulation was reflected in brain regions where the reward feedback is also encoded, such as the medial prefrontal cortex (MFC), precuneus, and posterior cingulate cortex. Furthermore, for the first time, we observed that the integration of the learning rate and the reward prediction error was represented in MFC activity. These findings extend our understanding of adaptive learning by demonstrating how it functions in a chain reaction of prediction updating.

## Introduction

The brain generalizes learned information to make predictions of the future. To improve the accuracy of these predictions, the learning process must incorporate new information to reflect the up-to-date states of the environment. The integration of new information involves two key factors: the prediction error that measures the discrepancy between current prediction and the observed environmental state, and the learning rate that determines to what degree the prediction error is applied to updating the prediction.

Prediction error has been thought to be calculated by the dopaminergic activity in the ventral tegmental area (VTA), then spreading to other brain regions via the afferent connections from the VTA (Schultz et al., 1997). Consistent with this theory, many functional magnetic resonance imaging (fMRI) studies have located brain structures in humans that reflect prediction error (for review, see Garrison et al., 2013). For example, D’Ardenne et al. (2008) detected activity related to the reward prediction error in the VTA of human participants. Outside the VTA, several researchers have also documented activity related to reward prediction error in regions that are connected to the VTA. Examples include subcortical structures such as the striatum (O’Doherty et al., 2003; Seymour et al., 2004; Pessiglione et al., 2006; Glascher et al., 2010; Jocham et al., 2011; Zhu et al., 2012; Eppinger et al., 2013) and nucleus accumbens (Niv et al., 2012). Cortical areas such as the medial prefrontal cortex (MFC) (Jocham et al., 2011; Eppinger et al., 2013) also appear to be involved.

A hallmark of learning is its flexibility, that is, the adaptive employment of prediction error in adjusting the prediction. This flexibility is attributed to the learning rate. To study this flexibility in adaptive learning, recent studies have employed computational models and algorithms to demonstrate how the learning rate shifts following changes in the environment (Behrens et al., 2007; Nassar et al., 2010; Payzan-LeNestour and Bossaerts, 2011; Jiang et al., 2014, 2015; McGuire et al., 2014). A common finding is that learning rate should favor recent information more, if there is change in the environment, both to reflect the up-do-date world state and to dampen the influence of outdated information. By contrast, if the environment is stable, learning rate should depend more on information sampled over an extended period of time (as opposed to recent information), so that learning is more robust against noise. In the brain, this change in the learning rate is associated with the anterior cingulate cortex (ACC) (Behrens et al., 2007), the anterior insula and adjacent inferior frontal gyrus (IFG) (McGuire et al., 2014; Jiang et al., 2015), and the MFC (McGuire et al., 2014).

However, many questions regarding the mechanisms of the learning rate remain unanswered. One question is whether other factors (besides volatility or rate of change in the environment) mediate the learning rate. In reward learning tasks, a possible candidate factor is reward feedback, known from past research to affect the subsequent strategy of humans performing a gambling task (Gehring and Willoughby, 2002; Yeung and Sanfey, 2004). Similarly, humans seem to use asymmetric learning rates for positive and negative prediction errors (Niv et al., 2012; Gershman, 2015). Another important, yet unanswered, question is how the learning rate and the prediction error are integrated to drive learning. In the reinforcement learning model (Rescorla and Wagner, 1972), the updating of prediction at time i + 1 (denoted as Δp_i+1_) is the prediction error (PE_i_) multiplied by the learning rate (*α_i_*) at time *i*; thus, Δp_i+1_ = α_i_ × PE_i_. Therefore, this joint effect of learning rate and prediction error on updating prediction can be tested as their interaction.

We hypothesized that: (1) the learning rate would be mediated by reward feedback; and (2) the integration of the learning rate and the prediction error would occur in the MFC, which is associated with both factors. To test these two hypotheses, we proposed a Bayesian model that provides computational mechanisms explaining how reward feedback influences the learning rate. Crucially, this model inferred the learning rate using the actual choices made by participants and the resulting reward, thus it accounts for individual differences and yield inference of the subjective learning rate. We further applied the model estimates to fMRI data, and provide new evidence of the neural substrates supporting these two hypotheses.

## Materials and Methods

### Subjects

Twenty-nine college students participated in this study. All participants had normal or corrected-to-normal vision. Two participants did not finish the task and were excluded from analysis, so the sample for behavioral analysis consisted of 27 participants (14 females, 20–24 years old, mean age = 22 years). In addition, four participants were excluded due to synchronization failure (the fMRI scanning and task did not start simultaneously) in at least one run, so the onsets of events in the behavioral task could not be mapped to the fMRI data. Data from two more participants were excluded due to normalization failure (i.e., SPM produced distorted normalized images after the normalization step; see below). Therefore, the final sample of fMRI analysis consisted of 21 participants (10 females, 20–23 years old, mean age = 22 years). This study was approved by the institutional review board of Chengdu University of Information Technology.

### Apparatus and Experimental Design

The task used in this study was programmed using Psychophysics Toolbox Version 3^1^. The stimuli (i.e., a red square and a green square) were displayed on a back projection screen. Participants viewed the display via a mirror attached to the head coil of the MR scanner and responded using two MR-compatible button boxes, one for each hand.

**FIGURE 1:**
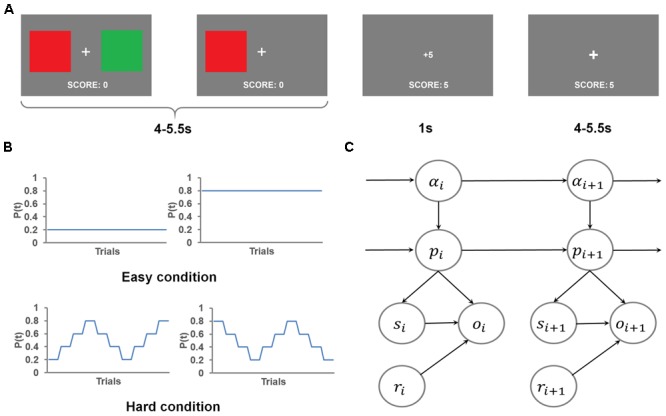
Task, experimental design and the graphical representation of the flexible learning model. **(A)** An example trial. Two color squares were presented on the screen for an interval, during which the red square was chosen, causing the green square being removed from the screen. The choice of the red square resulted in a gain of five points, which was displayed on the screen after the interval. The trial ended with a presentation of a fixation cross. **(B)** The four different time courses of the probability of getting high reward by selecting the red square used in this task. **(C)** The graphical representation of the generative model. Each node represents the state of a model variable. The edges show the flow of the information.

**Figure 1A** depicts the flow of events in the behavioral task trials. In each trial, participants chose between a red square and a green square to accumulate reward points, which determined the monetary reward they received after the task. At the beginning of each trial, a fixation cross appeared at the center of the screen, along with the two colored squares to the left and right of the cross for an exponentially jittered interval (4–5.5 s, step size = 0.5 s), during which the participants chose one square by pressing the button on the same side as the selected square. Once a response was made, the unselected square disappeared. After this interval, the reward gained (either 1 point or 5 points, corresponding to a low reward or a high reward, respectively) was presented at the center of the screen for 1 s, followed by the fixation cross shown for another exponentially jittered inter-trial interval (4–5.5 s, step size = 0.5 s), following which the stimulus display for the next trial appeared. If the participant did not respond, the trial was scored as *no response*. In case of no response (<0.3% of all trials), no points were gained and a message “+0” was shown. The lack of response in these trials precludes the inference of the mental states, therefore, the no response trials were excluded from behavioral and imaging analyses. The total points gained in the current run were displayed at the bottom of the screen throughout the task.

This task consisted of eight runs of 40 trials each. The participants were instructed to gain as many points as possible. Additionally, the participants were informed that at each trial, one color was more likely to lead to high reward than the other color. Further, we told participants that the more highly rewarded color would reset at the beginning of each run, and might change during the course of a run. Unbeknownst to the participants, at any trial, the sum of the two colors’ probabilities of getting a high reward was always 1. This constraint was used to keep the chance level of getting high reward at 50%. In order to create a wide range of high reward probabilities for the more rewarded color, we created two conditions (four runs for each condition, with the order of runs counterbalanced both within and across participants, **Figure 1B**): In a *hard* run (i.e., probability shifted within a run thus making the more rewarded color difficult to track), the underlying probability of getting a high reward by selecting the red square, changed every four trials, either in the order of 0.2, 0.4, 0.6, 0.8, 0.6, 0.4, 0.2, 0.4, 0.6, 0.8, or its reverse. In an *easy* run, this probability of getting high reward by selecting the red square remained fixed at 0.2 or 0.8 (two runs for each probability) throughout the run. This design ensured that the mean probability of getting high reward by selecting either color constantly was 0.5 (because the two colors were equally likely to be the more rewarding one) across this task.

### Procedure

All participants gave written informed consent before participating in the study. They then read the instructions for the task, performed a practice run to ensure that they understood the task, and underwent the scanning session. The scanning session consisted of one anatomical scan, eight runs of functional scans while the participants performed the task, one resting-state functional scan, and one diffuse tensor imaging (DTI) scan. The resting-state and DTI scans were not used in this study. After the scanning session, the participants received monetary compensation (a fixed amount for participation and a variable amount based on the score of a randomly selected run).

### Dynamic Analysis

In order to assess how trial history modulated future choices of color, and whether/how this modulation changed as a function of the reward received at the most recent trial, we conducted a response dynamic analysis (Lau and Glimcher, 2005). We started by dividing all trials into two sets, depending on whether the reward received at the previous trial was high or low. Subsequently, for each set, we constructed a linear model in the following form:

(1) sn = cn-1 sn-1 + cn-2sn-2

where s_n_, s_n-1_, and s_n-2_ represented the color choice (red = 1, green = -1) at trial *n, n*-1, and *n*-2, respectively. This model only considered the two most recent trials, because of the fact that, in hard runs, the reward probability changed every four trials. For each set, we used this model and behavioral data to estimate c_n-1_, and c_n-2_, which were the dependence of the choice at trial *n* on trial *n*-1 and *n*-2, respectively. To estimate c_n-1_, and c_n-2_, a design matrix with two regressors was constructed. The two regressors represented normalized trial-wise color choice in the past trial and two trials ago. This design matrix was then regressed against the vector encoding normalized trial-wise color choice to obtain estimates of c_n-1_, and c_n-2_. In the end, we compared the c_n-1_, and c_n-2_ estimates between the two sets (i.e., whether reward at trial *n*-1 was high or low).

### The Flexible Learning Model

To simulate how individuals learn the color that leads to better a chance of receiving high reward, we adapted the flexible control model that Jiang et al. (2014, 2015) have shown captures the flexible learning of control demand in a changing environment. This flexible learning model is also structurally similar to the model used by Behrens et al. (2007). In that study, subjects repeatedly bet on one of two options, only one of which leads to reward at each trial. The probability of reward may stay constant (stable condition) or flip (volatile condition). To simulate the task, the Behrens et al. (2007) model tracks the belief of the volatility (rate of change in reward probability) and the belief of reward probability. After each trial, the winning option is revealed to the model. Using this information the model updates the beliefs based on Bayesian inference. Crucially, the participants’ choices are not used in the model. In practice, even given the same trial sequence, different participants are likely to produce different patterns of behavior due to their different mental states (e.g., the belief of volatility and which option is better). However, in this case, due to the fact that the models in Behrens et al. (2007) and Jiang et al. (2014) do not use subjects’ behavior to infer the mental states, these models are unable to account for individual differences in behavior and will yield identical model estimates of mental states for all participants. To account for individual differences, the present model includes the participants’ choices of colors, which reflect the participants’ specific beliefs about the states of the task. Therefore, when two participants underwent the same trial sequence but produced different choices, the present model would consider the differences in choices and produce different model estimates.

The flexible learning model represented in **Figure 1C** has five variables, namely (1) the flexible learning rate, *α*, that quantifies the model’s (or a participant’s) belief concerning the relative weight of the most recent information (i.e., reward and choice of color observed) in learning; (2) the probability, *p*, of the red square leading to high reward; (3) observed selection of color, *s* (either 0 or 1, coded to correspond to green or red, respectively); (4) observed reward, *r* (either 0 or 1, coded to correspond to low or high reward, respectively), and (5) observed outcome, o, which is determined by *s* and *r* and encodes whether the selection resulted in the expected outcome (see below). The terms *s* and o are included to infer the hidden model belief states of *α* and *p*. The subscript *i* denotes the state of a variable at trial *i*. The dynamics of the distribution of learning rate across trials is defined such that the transition of the flexible learning rate is most likely to remain in its previous state; if it changes state, however, it is equally likely to jump to any other value, following a uniform probability distribution

(2) $$\begin{array}{c} p(\alpha_{i+1}|\alpha_{i})\;=\;1\;-\;k\;+\;k\delta \;(\alpha_{i+1}\;-\;\alpha_{i}) \end{array}$$

where *k* is the probability of the learning rate remaining the same as on the previous trial and 0 < *α_i_, k* < 1. δ(α_i+1_ -α_i_) equals to 1 if α_i+1_ is the same as α_i_ and equals to 0 otherwise.

Given the random sequencing of the task, it is not possible to make a precise prediction of the more rewarding color (e.g., predicting that the probability of the more rewarding color being red is 0.8 with 100% certainty, whereas this probability has 0 chance to be 0.799 or 0.801). Hence the prediction should be approximate, leading to a smooth distribution of *p_i_*. For example, a high likelihood of *p_i_* being 0.8 should also imply a high likelihood of *p_i_* at values close to 0.8. Hence, we used a propagation process to smooth the distribution of *p_i_* in the following manner:

(3) $$\begin{array}{c} v_{i+1}\;=\;\frac{1}{\alpha_{i+1}}\;-\;2 \end{array}$$

(4) $$\begin{array}{c} p_{i+0.5}\;\sim \;Beta\left(p_{i}v_{i+1}\;+\;1,\;v_{i+1}\;-\;p_{i}v_{i+1}\;+\;1\right) \end{array}$$

where p_i+0.5_ denotes the belief of the red color being the more rewarding color after the propagation process.

Up to this point, this model is identical to the flexible control model. The choices of equations and processing steps have been validated in Jiang et al. (2015). The following steps were conceptually similar to the flexible control model and were tailored to suit the current task. Specifically, after propagation, p_i+0.5_ is updated using a standard reinforcement learning rule with α_i+1_ playing the role of learning rate:

(5) $$\begin{array}{c} p_{i+1}\;\sim \;p_{i+0.5}\;+\;\alpha_{i+1}\;(o_{i}\;-\;p_{i+0.5}) \end{array}$$

where o_i_ denotes the outcome at trial *i*. Thus, o_i_ is 1 (i.e., supporting that the red color is associated with a better chance of obtaining high reward) when the chosen color is red and the reward is high, or when the chosen color is green and the reward is low. Otherwise, o_i_ is 0, indicating that the outcome does not support the red color being the more rewarding color. At this point, the expectation of distributions p(p_i+1_) and p(α_i+1_) are used, respectively, as estimates of the probability of the red square being the more rewarding one and the learning rate for behavioral and fMRI analyses.

When the actual selection and outcome were observed, the model beliefs were updated in the following manner:

(6) $$\begin{array}{c} p\left(k,\;\alpha_{i+1},\;p_{i+1}|s_{1},\;\ldots ,\;s_{i+1},\;o_{1},\;\ldots ,\;o_{i+1}\right)\\ \propto \;p\left(k,\;\alpha_{i+1},\;p_{i+1}|s_{1},\;\ldots ,\;s_{i,},\;o_{1},\;\ldots ,\;o_{i}\right)\\ {p(si+1, o}_{i+1}{|p}_{i+1}) \end{array}$$

where

(7) $$\begin{array}{c} p\left(s_{i+1},\;o_{i+1}|p_{i+1}\right)\;=\;p(o_{i+1}|s_{i+1},\;p_{i+1})p\left(s_{i+1}|p_{i+1}\right)\\ {=(1-|o}_{i+1}{- p}_{i+1}{|)(1 -|s}_{i+1}{- sp}_{i+1}|) \end{array}$$

In Eq. (7), |o_i+1_ -p_i+1_| quantified the discrepancy between estimated and actual outcomes; and |s_i+1_ - sp_i+1_| quantified the discrepancy between estimated and actual human behavior (i.e., actual selection of color, please see Eq. (8) for the definition of *sp*), which was then used to fit the model to human behavior in order to better infer the participant’s mental states and accounts for individual difference in reward learning. Therefore, p(S_i+1_, O_i+1_|P_i+1_) integrated the prediction error of the outcome and the prediction error of behavior (i.e., which color was selected), in order to infer the participants’ internal states.

Using Eqs. (6 and 7), we updated the joint distribution of k, α_i+1_, p_i+1_ with new observations s_i+1_, r_i+1_ and o_i+1_. This updated joint distribution was then fed into the simulation of the next trial (i.e., trial *i*+2).

To apply this model to simulate a participant’s behavior, the participant’s trial-by-trial color selections and the received reward were submitted to this model to generate trial-by-trial estimates of *α_i_* and *p_i_*. Specifically, the model maintained a joint distribution of p(k, α_i_, p_i_), which initialized as a uniform distribution at the beginning of each run. The distribution of a single variable, such as p(α_i_), could be calculated by marginalizing the joint distribution. At the beginning of trial *i*, we used Eqs. (2–5) to update p(k, α_i_, p_i_) and produce estimates of *α_i_* and *p_i_*. After the reward and the participant’s choice of color were observed, Eqs. (6 and 7) were applied to update the joint distribution of p(k, α_i_, p_i_). The simulation then entered the next trial. Note that in the flexible learning model, *α_i_* and *p_i_* represent random variables (i.e., probabilistic distributions). As mentioned above, in the following analyses, we only used the mean values of *α_i_* and *p_i_*. So, from this point on, we refer *α_i_* and *p_i_* to the mean of their corresponding random variables in order to keep the description of the methods and results concise.

After the trial-wise estimates of *α_i_* and *p_i_* were generated, the model’s prediction of the probability of selecting the red color at trial *i*, or ps_i_ was determined using the following softmax function:

(8) $$\begin{array}{c} ps_{i}\;=\;\frac{1}{1+e^{-(\beta_{1}\;+\;\beta_{2}p_{i})}} \end{array}$$

Where β_1_ and β_2_ were hyper parameters that were estimated by fitting ps_i_ to the actual choices of color made by each participant.

Similar to Jiang et al. (2015), the flexible learning model and the softmax function were estimated iteratively using an EM algorithm. This algorithm started with p = ps and estimated trial-wise α_i_ and *p_i_* based on Eq. (2–7) (E step). Then *s_i_* and the estimates of α_i_ and p_i_ were used to estimate ps_i_, β_1_ and β_2_ (M step), which were used in the E step in the next iteration. These two steps continued until the estimates of the hyper parameters converged. In this study, we implemented these procedures using Matlab. The scripts are available on request.

### Model Validation

To probe whether the flexible learning model accounted for behavioral data (i.e., actual color choices) better than typical, non-flexible reinforcement learning models, we performed model comparisons among four models: (a) the flexible learning model, (b) a reinforcement learning model with one fixed learning rate (RL_1 model), (c) a reinforcement learning model with one fixed learning rate for easy conditions and one for hard conditions (RL_2 model), and (d) a reinforcement learning model (PE-M) whose learning rate scales with the magnitude of prediction error (i.e., the learning rate is α|r - p|, where α is a base learning rate and |r - p| is the prediction error magnitude based modulation on α), which is similar to Pearce and Hall (1980). For models (b–d), the optimal learning rate(s) were obtained by an exhaustive search in the range of 0.01–0.99 (step size = 0.01) for each participant. Each reinforcement learning model was also connected to a softmax function to predict behavior. The free parameters in these softmax functions were estimated similarly to the softmax function in the flexible learning model. Thus, the flexible learning model had two free parameters (β_1_ and β_2_) for each participant; the RL_1, RL_2, and PE-M models had three (one learning rate plus β_1_ and β_2_), four (two learning rates and β_1_ and β_2_), and three (one base learning rate plus β_1_ and β_2_) free parameters, respectively. Thus, compared to the flexible learning model, the RL_1 and RL_2 models had one and two more free parameters (i.e., the learning rates), respectively.

In order to control for over-fitting and to provide generalizable results, we conducted cross-validation, which is a common practice in assessing learner performance in machine learning and multi-voxel pattern recognition. Specifically, we divided the runs into two-fold. For the flexible learning model and RL_1 model, each fold consisted of data from two easy runs and two hard runs; for RL_2 model, easy and hard runs were processed separately, so each fold had two runs from the same difficulty condition. To assess model performance, one-fold served as the training set to estimate the free parameters that best accounted for the training set. These estimated free parameters were then applied to the other fold (test set) to produce a trial-by-trial sequence of simulated probability of choosing the red color. Because the training and test sets were independent, this cross validation effectively reduced over-fitting. We repeated this procedure after exchanging the training and test sets. In the end, each model had a simulated trial-by-trial probability of choosing the red color. The performances of these simulations were then compared across models: For each model and each participant, we calculated the Bayesian information criterion (BIC) in the following manner:

(9) $$\begin{array}{c} \mathrm{BIC}\;=\;\mathrm{nln}\left(\;\sigma_{e}^{2}\right) \end{array}$$

where *n* is the number of trials and $\sigma_{e}^{2}$ is the error variance (Priestley, 1981) between model simulations and human behavior. Importantly, in calculating the BIC, we omitted the penalty for having additional free parameters, so that we only compared how well each model accounts for the behavioral data. It should be noted that this omission did not give the flexible learning rate any advantage in the model comparison, because it indeed had fewer free parameters than the other models. According to the definition of BIC, smaller prediction errors translated into lower BIC values, so the model with the lowest BIC had best performance.

### Statistical Analyses on Behavioral and Model Data

Repeated measure ANOVAs, two-tailed *t*-tests, and linear correlation analysis were conducted using SPSS or Matlab to analyze the behavioral and model data (see below for details).

### Image Acquisition

Images were acquired on a GE MR750 3.0T scanner. The anatomical images were scanned using a T1-weighted axial sequence parallel to the anterior-commissure-posterior commissure line. Each anatomical scan had 156 axial slices (spatial resolution = 1 mm × 1 mm × 1 mm, field of view = 256 mm × 256 mm, time repetition [TR] = 8.124 ms). The functional images were scanned using a T2^∗^-weighted single-shot gradient EPI sequence with a TR of 2 s. Each functional volume contained 43 axial slices (spatial resolution = 3.75 mm × 3.75 mm × 3.3 mm, field of view = 240 mm × 240 mm, TE = 28 ms, flip angle = 90°). Each fMRI run lasted for 416 s (208 TRs). During image acquisition, software monitored head movement in real-time. When the head movement exceeded 3 mm or 3° within a run, the scanning for that run was re-started using a new trial sequence.

### Image Analysis

The images were preprocessed using SPM12^2^. The first five volumes of each run were discarded before preprocessing. The remaining volumes were first realigned to the mean volume of the run, and went through slice-timing correction. The anatomical scan was co-registered to the mean volume, and then normalized to the Montreal Neurological Institute (MNI) template. The normalization parameters were applied to the slice-time corrected functional volumes, which were resampled to the spatial resolution of 3 mm × 3 mm × 3 mm). Finally, the resampled functional volumes were smoothed using a Gaussian kernel (FWHM = 8 mm).

We carried out a general linear model (GLM)-based analysis on the preprocessed fMRI data at each voxel for each individual (i.e., first-level analysis in SPM). This GLM consisted of up to 10 regressors, divided into three groups. The first group consisted of three regressors time-locked to the onset of the color squares at each trial: the stick function of each trial; *α* (the lack of a subscript indicates that this variable refers to the trial-by-trial time course of this variable); and the predicted reward probability of the chosen color (i.e., *p* or 1 -*p*, if the participant later chose red or green, respectively). We chose to time-lock *α* to the onset of the color squares to ensure that by that time the learning rate had been updated. The second group consisted of five regressors time-locked to the onset of the feedback at each trial: the stick function; the reward feedback, *r*; the signed reward prediction error, *pe* (*r - p* if red square was chosen, *r -* 1 + *p* if green square was chosen); the updating in learning, α × pe; and the α × r interaction that accounted for the behavioral pattern of post-high reward increase of learning rate (see below). The last group consisted of two regressors time-locked to the response at each trial: the stick function and the response (left or right). All regressors were concatenated across the eight runs of this experiment. The regressors were also normalized to remove the confounds from mean and magnitude. The time courses of model estimates (e.g., α, p) were obtained using the model parameters fit at the individual level. Therefore, the fact that the imaging analyses included fewer participants than the behavioral analyses did not change the model estimates for each participant.

To gauge the encoding strength of a variable (represented as a regressor), its regressor was first regressed against other regressors in the same group (i.e., sharing the same onset time) to remove shared variance so that the results could be uniquely attributed to the variable of interest. For example, to compute the encoding strength of *α*, the trial-by-trial time course of *α* was regressed against the other regressors in the same group. Then the post-regression *α* replaced the original *α* in the GLM.

This GLM was then convolved with SPM’s hemodynamic function and appended with nuisance parameters, including six head movement parameters (translations and rotations relative to *x, y*, and *z* axes) and grand mean vectors for each run to remove run-specific baseline fMRI signal. The resulting GLM was subsequently fit to the preprocessed fMRI data to estimate the coefficient for the variable of interest’s parametric modulator, which reflected the variable’s encoding strength (one estimate at each voxel of each individual’s data). To remove nuisance results at white matter and cerebrospinal fluid voxels, the statistical results were filtered using a gray matter mask obtained by segmenting the template and only keeping voxels with gray matter concentrations greater than 0.01. Finally, we conducted group-level *t*-tests on the estimates of encoding strength across participants to assess the group-level encoding strength (i.e., second-level analysis in SPM).

### Control for Multiple Comparisons

Statistical results were corrected for multiple comparisons at *P* < 0.05 for combined searchlight classification accuracy and cluster extent thresholds, using the AFNI ClustSim algorithm^3^. Specifically, 10,000 Monte Carlo simulations were conducted, each generating a random statistical map based on the smoothness of the map resulting from the group-level *t*-tests. For each randomly generated map, the algorithm searched for clusters using a voxel-wise *P*-value threshold of <0.001. The identified clusters were then grouped to produce a null distribution of cluster size. As a result, the ClustSim algorithm determined that an uncorrected voxel-wise *P*-value threshold of <0.001 in combination with a searchlight cluster size of 78–84 voxels (depending on the specific contrast) ensured a false discovery rate of <0.05.

## Results

### Behavioral Results

Twenty-seven participants performed the reward learning task (see Materials and Methods). At each trial, participants chose between a red square and a green square, and received either a high or a low reward based on the probability of high reward associated with the chosen color. Importantly, at each moment, one color had a better chance leading to a high reward than the other color (the sum of the two probabilities was always 1). In order to maximize reward, the participants must learn which color was more rewarding. To create a wide range of belief regarding the more rewarding color, the task contained two conditions. In the “easy” condition, the more rewarding color and its probability of yielding high reward remained constant at 80% throughout a run. Conversely, in the “hard” condition, the more rewarding color and its probability of obtaining high reward varied across time (from 20 to 80%, step size = 20%). Across the whole task, each color had a 50% chance of being the more rewarding color.

**FIGURE 2:**
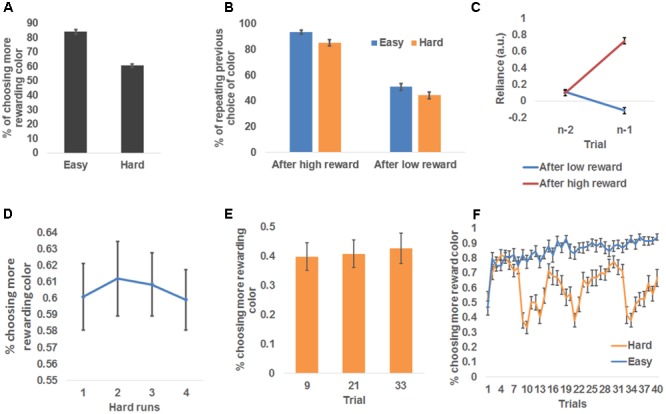
Behavioral results. **(A)** Group mean ± standard error of the percentage of choosing the more rewarding color, plotted as a function of run difficulty. **(B)** Group mean ± standard error of the percentage of repeating the previous trial’s choice, plotted as a function of reward magnitude and run difficulty. **(C)** Group mean and standard error of the dependence of the choice at trial *n* on choice history, plotted as a function of whether a high reward was received at trial *n*-1 and trial *n*-2. **(D)** Group mean and standard error of the percentage of choosing the more rewarding color, plotted as a function of the temporal order of hard runs. **(E)** Group mean and standard error of the percentage of choosing the more rewarding color, plotted as a function of the three trials (No. 9, 21, and 33) that immediately follow the change of the more rewarding color in hard runs. **(F)** Group mean and standard error of the percentage of choosing the more rewarding color, plotted as a function of trials in easy and hard runs separately.

Participants chose the more rewarding color more frequently than chance-level (i.e., 50%) for both conditions: easy condition: 84.0 ± 1.5%, *t*(26) = 23.05, *P* < 0.001, one-sampled *t*-test; hard condition: 60.5 ± 1.2%, *t*(26) = 9.12, *P* < 0.001, one-sampled *t*-tests (**Figure 2A**). Those outcomes indicate that participants followed the task instructions to learn the more rewarding color. Additionally, participants chose the more rewarding color more frequently in the easy condition than the hard condition, *t*(26) = 13.80, *P* < 0.001, paired *t*-test (**Figure 2A**).

To further probe how the participants adjusted their choice of color based on the reward received, we tested the frequency of the participants repeating their previous color choice. Because the more rewarding color was more likely to remain the same as on the previous trial than to switch to the other color, the participants should repeat their choices more frequently than chance level (50%). As expected, the overall frequency of choice repetition was significantly higher than chance [68.4 ± 1.8%, *t*(26) = 38.10, *P* < 0.001, one-sampled *t*-test]. To further test the differences in the frequency of choice repetition among experimental conditions, we conducted a repeated measure 2 (received reward: high, low) × 2 (difficulty: easy, hard) ANOVA (**Figure 2B**). This ANOVA revealed a significant main effect of received reward, *F*(1,26) = 291.74, *P* < 0.001, driven by a higher frequency of repeating color choice after receiving a high reward (89.3 ± 1.8%) than after receiving a low reward (47.6 ± 2.6%), suggesting that high reward enhanced the participants’ belief that the selected color was the more rewarding one. The main effect of difficulty was also significant, *F*(1,26) = 69.37, *P* < 0.001, driven by higher likelihood of repeating color choice in easy condition (72.2 ± 1.8%) than hard condition (64.7 ± 2.0%). This difference possibly reflected the fact that the more rewarding color changed more frequently in hard than easy condition. The reward type × difficulty interaction was not significant [*F*(1,26) = 0.66].

We also conducted an additional dynamic analysis (Lau and Glimcher, 2005) that compared how the choice of color at trial *n* relied on the interaction between trial history and the reward received at trial *n-*1 (Browning et al., 2015). The results are shown in **Figure 2C**: although the contribution of color choice at trial *n-*2 to color choice at trial *n* did not vary as a function of reward feedback at trial *n*-1, *t*(26) = 0.31, paired *t*-test, the contribution of color choice at trial *n-*1 differed significantly between reward feedback levels, *t*(26) = 17.16, *P* < 0.001, paired *t*-test. Specifically, when receiving a high reward at trial *n-*1, color choice at trial *n-*1 had a positive influence (i.e., promoting choice repetition) on the choice at trial *n*, reliance: 0.72 ± 0.04, *t*(26) = 20.30, *P* < 0.001, one-sample *t*-test, whereas this influence became negative if the reward was negative (i.e., promoting choice change), reliance: -0.12 ± 0.04, *t*(26) = -3.00, *P* = 0.006, one-sample *t*-test. This analysis again confirmed that the reward received at the current trial modulated how likely the choice would be repeated at the forthcoming trial.

In hard runs, it may be possible that the participants became aware of the change patterns of the rewarding probability and proactively altered their learning rate to adapt to the change. If this were true, task performance in hard runs should increase with time on task. We conducted a repeated-measures one-way ANOVA on the four hard runs’ probability of choosing the more rewarding color, and did not find a significant change of performance across runs, *F*(3,24) = 0.08 (**Figure 2D**). Furthermore, we tested the effect of within-run learning of change patterns. To this end, we compared the probability of choosing the more rewarding colors among the three trials (No. 9, 21, and 33; **Figure 1B**) that are immediately after the change of the more rewarding color (i.e., *p* changed from 0.4 to 0.6 or vice versa). If the participants learned the change patterns and proactively adjusted to the change, we expected an increase in the probability of choosing the more rewarded color across the three trials. However, a repeated-measures one way ANOVA did not find any effect, *F*(2,25) = 0.08 (**Figure 2E**). Collectively, the participants appeared not to be able to apply the change patterns to boost their performance in this task.

A closer look at the time course of the participants’ choices (**Figure 2F**) showed that, in easy runs, the probability of choosing the more rewarded color kept increasing, suggesting the participants readily learned the underlying reward probability. However, the probability of choosing the more rewarded color fluctuated in hard runs, due to the change of the underlying reward probability. A general trend in the hard condition was a sudden drop in the ability to identify the more rewarded color following the change of the more rewarded color (i.e., after trials #9, 21, and 33, similar to **Figure 2E**), and a gradual recovery that suggests that the participants continued to learn the current more rewarded color.

### Model Comparison

**FIGURE 3:**
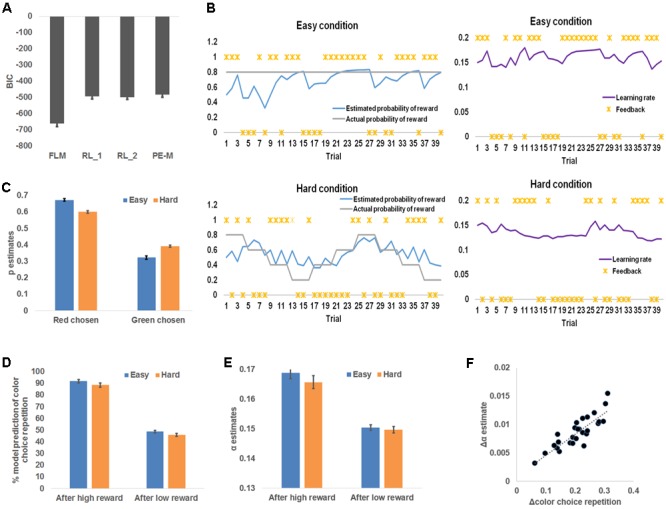
Model-based behavioral results. **(A)** Model comparison results. From left to right, each bar represents group mean Bayesian information criterion (BIC) and mean standard error of the flexible learning model (FLM), reinforcement learning model with one (RL_1) learning rate and two (RL_2) learning rates (one for each level of difficulty), and a reinforcement learning model whose learning rate scales with the magnitude of prediction error (PE-M). **(B)** Sample time courses of the learning rate and the expected probability that high reward for the red square. The locations of asterisks indicate high or low reward received at each trial. **(C)** Model belief that red was the more rewarding color, plotted as a function of difficulty and the chosen color. **(D)** Model simulation of the percentage of choice repetition, plotted as a function of reward magnitude and difficulty. **(E)** Model belief of the learning rate, plotted as a function of reward magnitude and difficulty. **(F)** Individual increments of the probability of choice repetition (following high reward minus following low reward), plotted against the individual increments (following high reward minus following low reward) of the model estimates of the learning rate. The dotted line depicts the trend line.

In order to model human behavior in this task and to infer latent learning related states, we employed a flexible learning model (see Materials and Methods) that self-adjusts the learning rate, α, and the belief that the red color was the more rewarded color, *p*, based on observed color choice and received reward. To ascertain that this model accounted for the behavior better than conventional reinforcement learning models, we conducted a model comparison analysis among the flexible learning model and reinforcement learners with one fixed learning rate (RL_1), reinforcement learners with two fixed learning rates (RL_2, one learning rate for each difficulty condition), and reinforcement learners whose learning rate scales with the magnitude of prediction error (PE-M). The flexible learning model accounted for trial-by-trial color choice best in the four models (i.e., the flexible learning model had the lowest BIC) for all participants (**Figure 3A**). Therefore, we concluded that the flexible learning model explained variance in human behavior better than conventional reinforcement learners in this task. Consequently, we focused on the flexible learning model in the following analyses.

### Model-Based Behavioral Analysis

The flexible learning model generated trial-by-trial estimates of α and *p*, which drove the learning of the more rewarding color (**Figure 3B**). To test whether these model estimates reflected behavioral patterns, we first conducted a repeated measures 2 (chosen color: red, green) × 2 (difficulty: easy, hard) ANOVA on the *p* estimates (**Figure 3C**). That analysis revealed a significant main effect of color choice, *F*(1,26) = 507.48, *P* < 0.001, whereby red square choices were associated with higher *p* estimates (i.e., stronger belief that red was the more rewarding color) than green square choices, with *p* estimates in red square choices: 0.64 ± 0.01 and estimates in green square choices: 0.36 ± 0.01. This result matches the model’s specification that *p* was associated with the red square. In other words, this result showed that the participants tended to choose the more rewarded color predicted by the model. Additionally, we discovered a significant interaction between chosen color and difficulty, *F*(1,26) = 177.49, *P* < 0.001, driven by a smaller effect of chosen color on *p* estimates in hard (0.35 ± 0.01) than easy (0.21 ± 0.01) conditions. This reduced effect may reflect the fact that the more rewarded color was more difficult to learn in the hard condition. The main effect of difficulty was not significant. Next, to confirm that *p* guided the prediction of chosen color in the right direction, we found that the responsible parameter β_2_ (see Materials and Methods) was greater than 0 for all participants (i.e., higher *p* leads to higher probability of choosing the red square; range: 4.5–25.0).

Recall that participants repeated the choice of color more frequently following a high reward than a low reward. The flexible learning model accounted for this result in two ways: First, the pattern of simulated probability of repeating the previous choice, calculated by applying trial-based *p* estimate to Eq. (8), then comparing the result to the previous choice, should resemble **Figure 2B**. Second, the model accounts for this observation by increasing *α* after high reward to augment the current selection’s influence on selecting (the same) color in the next trial. To test these model predictions, we conducted separate 2 (received reward: high, low) × 2 (difficulty: easy, hard) ANOVAs on the probability of repeating the previous choice (**Figure 3D**) and *α* estimates (**Figure 3E**). Similar to the behavioral results, the first ANOVA yielded a significant main effect of both reward type, *F*(1,26) = 384.22, *P* < 0.001, driven by a higher likelihood of repeating a color choice after high reward (90.0 ± 1.7%) than low reward (47.2 ± 1.2%); and a main effect of difficulty, *F*(1,26) = 20.11, *P* < 0.001, driven by a higher likelihood of repeating color choice after an easy condition (70.2 ± 1.4%) than hard condition (67.1 ± 1.5%). The interaction was not significant [*F*(1,26) = 0.19].

Also consistent with the model prediction, the second ANOVA revealed a main effect of reward type, *F*(1,26) = 265.37, *P* < 0.001; learning rate estimates were higher after high reward (0.167 ± 0.002) than low reward (0.150 ± 0.001). No other effects were significant, *F*(1,26) < 2.76, *P* = 0.11. As a control analysis, we also tested whether the prediction error of reward is a better predictor than the magnitude of reward for selecting the same response in the next trial, given their high correlation. To this end, we used a binary vector to represent whether the color selection was repeated in the next trial for each participant; and compared how much variance in this vector could be explained by trial-wise reward magnitude vs. trial-wise prediction error of reward. For all participants, reward magnitude was a better predictor than prediction error (additional variance explained by reward magnitude ranged from 2.5 to 13.5%). The results supported the notion that reward magnitude is a more likely driving factor for repeating selection than reward prediction error.

Finally, to test how *α* accounted for individual differences in repetition of color choice, we conducted the linear correlation analysis between the increase of *α* (high reward – low reward) and the increase of the frequency of choice repetition (high reward – low reward) across participants, and found a strong positive linear correlation (*r* = 0.86, *P* < 0.001; **Figure 3F**). Note that in Eq. (2), we did not constrain which way the learning rate should go conditioned on the type of reward (high or low) received (i.e., the model is not pre-defined to show the increase of learning rate following high reward). Furthermore, the same model was fit to individual behavior data. Therefore, the individual difference in the amount of learning rate increase following high reward is solely attributable to the participants’ behavior. In other words, the results in **Figure 3F** indeed indicate that the change in the likelihood of choice repetition was captured by the change in learning rate estimates. Given that the flexible learning model takes the participants’ choices of colors as input in order to account for individual differences, this model revealed individual differences in raising the learning rate following a high reward in relation to a low reward. According to the reinforcement learning algorithm, the learning rate is also the weight of the current choice on determining the next choice. Therefore, participants’ increasing the learning rate more after a high reward than a low reward will let a choice that led to high reward have more weight on determining the next choice (i.e., more likely to repeat the choice), as compared to a choice that led to low reward, thus producing the correlation in **Figure 3F**.

Taken together, these results suggested that the flexible learning model estimates of *α* and *p* captured the behavioral patterns. Therefore, the model provided meaningful learning-related information for the following imaging analyses.

### Imaging Results

**FIGURE 4:**
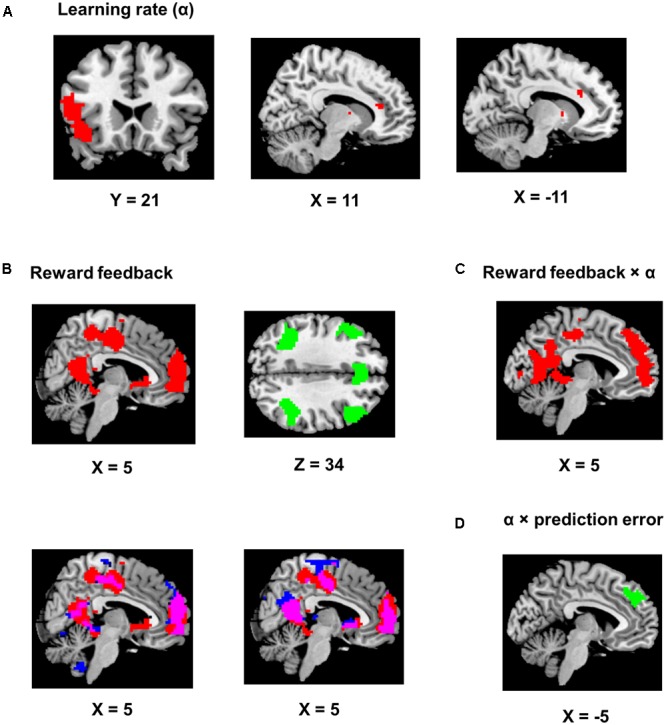
Imaging results. Positive encoding and negative encoding are shown in red and green, respectively. The ch2bet template from mricron was used to be the reference brain to make figures from the statistical maps. **(A)** Encoding of the learning rate. The left panel shows the IFG/anterior insula region (*P* < 0.05, corrected). The two other panels show the dACC clusters (*P* < 0.001, uncorrected). **(B)** Encoding of the reward feedback (*P* < 0.05, corrected). The lower left panel shows the overlap (in purple) of the clusters in **(A)** and brain regions showing significantly stronger encoding of reward feedback than reward prediction error (in blue). The lower right panel shows the overlap (in purple) of the clusters in **(A)** and brain regions significantly encoding of reward feedback when the reward prediction error regressor was also included in the GLM (in blue). **(C)** Brian regions showing significant positive reward feedback × learning rate interaction (*P* < 0.05, corrected). **(D)** An MFC region showing significant learning rate × reward prediction error interaction (*P* < 0.05, corrected).

Using trial-by-trial model estimates derived from the flexible learning model, we examined the encoding of model variables and their interactions in the brain, based on data obtained from fMRI scans while the participants performed the task (**Table 1**). We found that the learning rate reliably co-varied with fMRI signals in the left IFG and adjacent anterior insula (*P* < 0.05, corrected; peak MNI coordinates: -51, 17, 11, **Figure 4A**). Although not statistically significant after controlling for multiple comparisons, three scattered ACC clusters (size: 7–13 voxels) showed encoding of the learning rate at the *P* < 0.001 (uncorrected) level (**Figure 4A**). Given that the learning rate in the flexible learning model reflected the volatility in the task, these ACC clusters were consistent with earlier findings from Behrens et al. (2007). No other clusters surpassed significance threshold.

**Table 1:** Summary of fMRI results.

Location	Peak MNI	Peak *t*-value	Cluster size (#voxels)
**High reward > low reward**			
Middle Cingulate Gyrus, Posterior Cingulate Gyrus, Precuneus	(–15, –49, 11)	11.16	787
Medial Superior Frontal Gyrus	(0, 56, 17)	11.18	771
R. Precentral Gyrus, R. Rolandic Oper	(33, –13, 38)	9.26	604
L. Hippocampus, L. Parahippocampal Gyrus, L. Fusiform Gyrus	(–33, –37, –16)	9.36	555
R. Middle Temporal Gyrus, R. Superior Temporal Gyrus	(57, 2, –10)	9.26	548
L. Middle Temporal Gyrus, L. Superior Temporal Gyrus	(–51, –7, –7)	8.93	526
R. Hippocampus, R. Parahippocampal gyrus, R. Fusiform Gyrus	(36, –22, –7)	10.28	449
R. Precentral Gyrus, R. Postcentral Gyrus, R. Rolandic Oper	(–45, –10, 20)	9.15	360
L. Superior Occipital Gyrus, L. Middle Occipital Gyrus	(–45, –79, 17)	7.77	338
R. Superior Occipital Gyrus, R. Middle Occipital Gyrus	(33, –91, 8)	7.50	218
**Negative encoding of reward feedback**			
L. Inferior Parietal Gyrus	(–45, –49, 44)	–7.21	306
L. Medial Superior Frontal Gyrus	(–6, 26, 41)	–8.7	291
R. Middle Frontal Gyrus	(48, 29, 35)	–6.38	282
R. Supramarginal Gyrus	(45, –40, 44)	–6.97	247
L. Middle Frontal Gyrus	(–48, 29, 32)	–7.77	106
L. Insula	(–30, 26, –7)	–8.43	92
R. Insula	(33, 23, –4)	–7.44	90
**Interaction between feedback and reward prediction error**			
L. Orbital medial Frontal Gyrus, L. Medial Superior Frontal Gyrus	(–3, 62, –7)	6.02	914
R. Precuneus, R. Posterior Cingulate, R. Fusiform Gyrus	(30, –37, –16)	4.99	334
L. Fusiform Gyrus, L. Lingual Gyrus, L. Parahippocampal Gyrus	(–27, –61, –4)	6.50	162
R. Superior Temporal Gyrus, R. Middle Temporal Gyrus	(51, –55, 5)	4.98	150
L. Middle Temporal Gyrus, L. Inferior Temporal Gyrus	(–48, –13, –16)	5.06	117
R. Rolandic Oper, R. Precentral Gyrus, R. Postcentral Gyrus	(69, –10, 14)	5.09	98

*Thresholds were set at *P* < 0.05, multiple comparisons corrected. MNI, Montreal Neurological Institute; L., left; R., right.*

The reward feedback was represented both positively (i.e., higher brain activity if reward was higher than expected) and negatively (i.e., higher brain activity if reward was 1 point) in the brain. Specifically, positive encoding of the feedback was revealed in a widespread brain network, most significantly in the MFC (*P* < 0.05, corrected; peak MNI coordinates: 0, 56, 17, **Figure 4B**) and the precuneus and posterior cingulate cortex (PCC, *P* < 0.05, corrected; peak MNI coordinates: -15, -49, 11, **Figure 4B**), whereas negative encoding of the reward feedback was primarily found in the attentional control networks (**Figure 4B**; all regions reported had *P*s < 0.05 after correction for multiple comparisons), including dorsal ACC (peak MNI coordinates: -6, 26, 41), bilateral insular and surrounding IFG (peak MNI coordinates: -30, 26, -7; 33, 23, -4), bilateral middle frontal gyri (peak MNI coordinates: -48, 29, 32; 48, 29, 35), and bilateral inferior parietal lobules (peak MNI coordinates: -45, -49, 44; 45, -40, 44).

An alternative explanation was that these regions encoded the reward prediction error, which was correlated with the reward feedback. This is because a high reward always produces positive prediction error and a low reward always produces negative prediction error. One way to tease apart the contribution of reward feedback from the contribution of prediction error would be to regress them against each other and compare the encoding strength of the residues. However, this regression may result in strong anti-correlation between the residues and confound the results. Therefore, as a control analysis, we constructed two GLMs based on the original GLM, with one excluding the reward prediction error regressor and the other excluding the reward feedback regressor, and compared the encoding strength (i.e., beta estimates) of the reward feedback with the encoding strength of the reward prediction error throughout the brain. By separating the regressors in different GLMs, we addressed the anti-correlation issue by not regressing reward feedback and prediction error against each other. The reward feedback regressor displayed stronger encoding strength than the reward prediction error regressor mostly in brain regions showing positive encoding of the reward feedback (**Figure 4B**, lower left panel, *P* < 0.05, corrected). Moreover, when both reward feedback and reward prediction error were included in the same model (again without regressing them against each other) for direct comparison, the reward feedback still displayed significant encoding strength in these regions (**Figure 4B**, lower right panel, *P* < 0.05, corrected), whereas the reward prediction error did not show significant encoding strength. On the other hand, there was no significant difference in encoding strength in the regions showing negative encoding of the reward feedback.

The key imaging analyses in this study concern how the flexible learning rate interacts with feedback and reward prediction error. The behavioral analysis revealed increased learning rates following high reward. Accordingly, we tested the interaction between the updated learning rate and the reward feedback. Given the positive interaction (i.e., high reward associated with high learning rate) found in the behavioral data, we also expected this interaction to be positive in the fMRI data. The results showed that the MFC (*P* < 0.05, corrected; peak MNI coordinates: -3, 62, -7, **Figure 4C**) and the precuneus and PCC (*P* < 0.05, corrected; peak MNI coordinates: 30, -37, -16, **Figure 4C**) reported above also showed positive learning rate × feedback interaction. Note that our analysis removed variance shared between model estimates and interactions prior to imaging analysis, so the overlapping results reported are unlikely to be attributed to similarity between model variables and their interaction terms. Finally, we performed an fMRI analysis seeking brain regions encoding the learning rate × prediction error interaction, and found that only a left MFC region negatively encoded this interaction term (*P* < 0.05, corrected; peak MNI coordinates: -3, 47, 32, **Figure 4D**).

## Discussion

The brain adaptively adjusts its learning rate to incorporate recent information in order to make more precise predictions of the future. To address the question of how the learning rate is adjusted, we applied a computational model based on the reinforcement learning model with a flexible learning rate to account for human behavior and brain activity in a reward learning task. Unlike other studies (Niv et al., 2012) that traced the reward probability for each option, the present study focused on learning which option is more rewarding. This difference of modeling strategy is because, regardless of the manipulation of rewarding probability for individual options, the optimal strategy for the task is always to select the most rewarding option. From this perspective, a novel finding in behavioral analysis was that, in the learning of the more rewarding color, the reward magnitude modulated the learning rate, which further predicted a greater likelihood that participants would repeat a previous choice after obtaining high reward than low reward. In subsequent fMRI analyses, this learning rate × outcome interaction was found in brain regions where the reward feedback was also encoded. Furthermore, to address how the learning rate mediates learning, we probed the representation of the learning rate × prediction error interaction, and found that the trial-by-trial fluctuation in this interaction correlated with the fMRI activity in the MFC.

We started by validating the proposed flexible learning model. Behavioral data was better explained by this model, as compared to reinforcement learning models assuming fixed learning rates (**Figure 3A**). This suggested that participants indeed adjusted the learning rate during the task (**Figure 3F**). The result that the flexible learning model outperformed a reinforcement learning model whose learning rate scales with the magnitude of prediction error also suggests that the change of learning rate is not simply mediated by prediction error alone. The estimates of learning rate can be influenced by response history and the interaction between the choice of color and reward (e.g., repeating previous choice after high reward). This integration also allows the model to account for individual differences (**Figure 3F**).

The finding that the condition-mean learning rate did not differ significantly between the easy and hard conditions suggested that the learning rate adapted to changes at the trial level rather than in a more tonic way (i.e., the block level). This result is also consistent with the flexible learning model‘s design principle of feedback-driven, trial-by-trial level learning. Previous studies have shown higher estimate of learning rate (volatility) in faster changing environment (Behrens et al., 2007; Jiang et al., 2015). This study revealed additional contribution to the learning rate from reward magnitude. Because the subjects obtained higher reward in more frequently in easy condition than hard condition, learning rate may be boosted higher in easy condition. Taken together, the lack of difference in the learning rate between easy and hard conditions may be a result of the modulation of volatility and reward canceling out each other.

The fMRI analysis showed that the learning rate was encoded in the ACC and the IFG (**Figure 4A**). The former finding replicated the Behrens et al. (2007) study, which documents that volatility (high volatility translated into high learning rate; see Jiang et al., 2015 for details) is encoded in the ACC. The other finding, that the learning rate was encoded in the IFG, echoes previous studies demonstrating that the IFG activity reflects strategy changes in reading (Moss et al., 2011) and memory-encoding (Cohen et al., 2014). Additionally, the IFG is involved in affective switching (Kringelbach and Rolls, 2003; Remijnse et al., 2005), and task switching (Crone et al., 2006). The IFG finding in the present study is also in line with the involvement of the IFG in shifting learning rate/strategy.

A main finding in the behavioral results was that the participants tended to choose the same color more often after receiving a high reward than a low reward (**Figure 2B**). Given the fact that the participants learned the more rewarding color and chose it more often than chance level (**Figure 2A**), it is likely that this difference of choice repetition is (in part) due to the larger prediction error from unexpected low reward. Additionally, this change may also be attributed to the learning rate, which influences the updating of the prediction and hence the choice at the next trial. This hypothesis was tested using the flexible learning model, which treats both prediction error and the learning rate as variables that can change after each trial. Like the probability of choice repetition, the model estimate of the learning rate in the subsequent trial increased with reward magnitude. Moreover, the amount of increment in the learning rate also predicted the increase of choice repetition across participants, thus providing strong evidence that the learning rate served as a mechanism leading to the more frequent choice repetition after high reward. An alternative explanation is that the increased prediction error magnitude that signaled the importance of this trial led to the increase in the learning rate. However, this explanation was not supported in that the learning rate was indeed lower after low reward, when the prediction error magnitude should be high (because the participants successfully tracked the more rewarding color most of the time; **Figure 2A**). According to the flexible learning model, increasing the learning rate after receiving high reward would increase the influence of the current high reward on future predictions of the more rewarding color. As a result, the current color that yielded high reward would be more likely to be selected than if a low reward were received at the current trial. This finding is also related to the literature of the exploration–exploitation tradeoff in reward learning (Cohen et al., 2007), in that high reward is more likely to result in exploitation (i.e., repeatedly choosing the same color to accumulate high reward).

To locate the neural substrates that support feedback mediation of the learning rate, we first conducted an fMRI analysis to test the encoding of the reward feedback. Large-scale encoding of the feedback was observed (**Figure 4B**), including both positive (i.e., high reward > low reward) encoding in the MPFC, precuneus and the PCC, and negative (i.e., low reward > high reward) encoding in the control network. Activity in these regions is highly consistent with the findings reported in Eppinger et al. (2013), who studied brain activity associated with positive learning and negative learning. Specifically, positive encoding of reward feedback may reflect reward valuation in the brain (Grabenhorst and Rolls, 2011), whereas negative encoding may suggest the engagement of the control network in error processing (e.g., obtaining a low reward while anticipating a high reward may be considered as an “error”) or performance monitoring (Botvinick et al., 2004), or both. Given the high correlation between the feedback and the reward prediction error, we performed an additional control analysis by comparing the encoding strength between the feedback regressor and the reward prediction error regressor throughout the brain, while keeping other regressors in the GLM. The feedback regressor displayed stronger encoding strength in all the aforementioned regions showing positive encoding of reward feedback, implying a higher likelihood of the feedback being encoded than the reward prediction error (**Figure 4B**). Moreover, when we included both regressors of reward feedback and reward prediction error in the same GLM, only the former showed significant encoding in the reported regions (**Figure 4B**). Therefore, the reported regions in **Figure 4B** were more likely to represent reward feedback than reward prediction error.

Our reported regions did not include striatum, which has been shown by other research to encode prediction error (O’Doherty et al., 2003; Seymour et al., 2004; Pessiglione et al., 2006; Glascher et al., 2010; Jocham et al., 2011; Zhu et al., 2012; Eppinger et al., 2013). We speculate that this lack of striatum finding is because, as we have shown, these regions were likely to encode reward feedback rather than reward prediction error. Although reward feedback and reward prediction error were highly correlated, the latter was defined as the difference between reward feedback and the prediction from the learning model. Therefore, reward feedback, as compared to reward prediction error, may be less involved in learning and prediction, and in turn less likely to be represented in the striatum that supports prediction from learning (Jiang et al., 2015).

We then performed an independent analysis seeking the brain regions encoding the interaction between the reward feedback and the learning rate that integrated this feedback (via prediction error), and found high degree of overlap between regions encoding this interaction and regions positively encoding the reward feedback (**Figure 4C**). Moreover, these results shown in **Figure 4C** were obtained after removing the shared variance with these two variables, thus excluding the confound of their correlation. Interestingly, the direction of the interaction in these regions was also in line with the positive encoding of reward feedback (**Figure 4C**), which further suggested that the feedback played an important role in mediating the learning rate in these regions. Therefore, these results, when combined together, strongly supported the notion that the learning rate was updated as the feedback was processed.

In a final set of analyses, we examined the integration of the learning rate and the reward prediction error, and discovered an interaction between the learning rate and the reward prediction error in the MFC. Surprisingly, that interaction is negative: For example, when keeping the learning rate constant, fMRI activity decreased as the reward prediction error increased. Nevertheless, an explanation for this negative interaction was that prediction error could be encoded reversely by MFC neurons that signaled negative prediction error (e.g., receiving low reward while high reward was expected: Amiez et al., 2006; Matsumoto et al., 2007; Oliveira et al., 2007; Jessup et al., 2010). Consistent with this explanation, our fMRI results showed higher activity in the ACC (which is usually considered as part of the MFC) for low reward feedback (corresponding to negative prediction error, **Figure 4B**). Moreover, (Jessup et al., 2010) demonstrated that fMRI activity in the MFC is higher when participants lose in a trial in a gambling task. Interestingly, higher MFC activity when losing only occurs when participants were more likely to win than lose, which was exactly the case in the task used in the present research (**Figure 2A**). Similar findings showing adaptive learning have also been reported in other domains of learning. For example, based on a multi-level Bayesian model that accounts for multiple forms of uncertainty (Mathys et al., 2011; Iglesias et al., 2013) showed that prediction error is modulated by perceptual precision in sensory learning. Moreover, Iglesias et al. (2013) also reported activity in an MFC region, similar to that illustrated in **Figure 4D**, that encodes precision-modulated prediction error.

In addition to showing that the integration of learning rate and prediction error co-varies with neural signals in MFC, Chien et al. (2016) demonstrated that fMRI signals from two striatum regions that separately represent learning rate and reward prediction error jointly account for fMRI signals in the ventral MFC, in which the reward prediction was represented. This finding further links the integration of learning rate and prediction error to the updating of reward prediction. Published results and our own collectively suggest that MFC may serve the function of modulating prediction error-driven updating of future predictions across different domains of learning.

The MFC is involved in multiple cognitive functions, such as error and control monitoring (Botvinick et al., 1999, 2001, 2004), speed-accuracy tradeoff (Yeung and Nieuwenhuis, 2009), reward learning (Rushworth and Behrens, 2008; Jessup et al., 2010), decision making (Rushworth et al., 2004), and social cognition (Amodio and Frith, 2006). Recent modeling work that attempts to summarize the role of the MFC in those functions stresses the importance of learning in MFC functions (Alexander and Brown, 2011; Silvetti et al., 2011). In particular, prediction error is considered as the driving force of learning. Our findings further extend this notion by showing that (a) the MFC encoded the modulation of the reward feedback on the learning rate, and (b) the MFC is also involved in adaptive learning, such that the impact of the prediction error on learning can be flexibly adjusted based on factors such as the reward feedback and the volatility (i.e., rate of change in the environment). Therefore, the learning mechanism can determine how important and reliable (or both) the new piece of information is, and adjusts its weight in the new prediction accordingly.

The fMRI activity patterns showing representation of reward magnitude and its interaction with the learning rate in MFC and PCC overlap the default mode network. We speculated that this overlap may be related to a possible function of the default mode network of supporting internal simulations while ignoring external stimulation (Buckner et al., 2008). That is, the default mode network may be more engaged to support the simulations of performing future trials after high reward and when both reward and learning rate were high.

We employed two conditions with different degrees of difficulty in this task. The difficulty manipulation affected both behavioral (**Figure 2B**) and simulation (**Figures 3C,D**) data. Consequently, could difficulty be a confounding factor for the results reported? A closer look at the experimental task revealed that task difficulty consisted of two aspects: First, the hard condition included both a change of the more rewarding color and its rewarding probability. In the flexible learning model, this difference was accounted for by the flexible learning rate that adapted to the change in the environment. Second, the hard condition included additional rewarding probabilities of 0.4 and 0.6, which were, by definition, more ambiguous than probabilities of 0.2 and 0.8 in inferring which color was more rewarding. In other words, probabilities of 0.4 and 0.6 would generate larger reward prediction errors than probabilities of 0.2 and 0.8. Reward prediction error was also modeled by the flexible learning model. Therefore, both aspects of difficulty manipulation in this task have been accounted for. In other words, in this task, we attempted to use learning models to quantify and integrate the effects of abstract factors such as difficulty and change in the environment, so that behavior and neural activity patterns may be parsimoniously explained by concrete, quantifiable factors such as learning rate and reward prediction error.

One caveat of this study is that the task only used two levels of reward magnitude, which resulted in correlation between reward magnitude and reward prediction error. In addition, given that the goal of this study is to maximize reward, obtaining a low reward can be seen as the outcome of an “incorrect” response, which may further drive learning through prediction error. Even though we alleviated this confound by conducting additional behavioral and fMRI control analyses to show that reported results were better explained by reward magnitude than reward prediction error, an experimental design that varies reward magnitude on a trial-by-trial basis can de-correlate these two factors in the first place, and thus would be a better option than a design with only high vs. low reward.

## Conclusion

Our findings provide novel evidence suggesting that, in order to achieve the goal of accumulate more reward, the reward feedback (high or low reward) mediated learning rate; and the learning rate further drove the reward prediction error to update the future decision. Importantly, the MFC seems to underlie both functions.

## Ethics Statement

This study was carried out in accordance with the recommendations of Institutional Review Board of Chengdu University of Information Technology with written informed consent from all subjects. All subjects gave written informed consent in accordance with the Declaration of Helsinki. The protocol was approved by the Institutional Review Board of Chengdu University of Information Technology.

## Author Contributions

XW, DZ, and JZ designed research. XW, TiW, CL, TaW, and JJ analyzed data. CL acquired the data. XW, TiW, JJ, and JZ wrote the paper. DZ, JJ, and JZ revised the draft.

## Conflict of Interest Statement

The authors declare that the research was conducted in the absence of any commercial or financial relationships that could be construed as a potential conflict of interest.

## Footnotes

1 http://psychtoolbox.org/

2 http://www.fil.ion.ucl.ac.uk/spm/

3 http://afni.nimh.nih.gov/pub/dist/doc/program_help/3dClustSim.html
